# Oral health promotion practices of Australian community mental health professionals: a cross sectional web-based survey

**DOI:** 10.1186/s12903-021-01438-x

**Published:** 2021-02-25

**Authors:** Roisin McGrath, Rodrigo Marino, Julie Satur

**Affiliations:** The Melbourne Dental School, The University of Melbourne, Melbourne, VIC 3053 Australia

**Keywords:** Mental health, Oral health promotion, Mental health support workers, Australia

## Abstract

**Background:**

This study explored the oral health promotion practices of Australian community mental health professionals working with people living with severe mental illness (SMI).

**Methods:**

An anonymous cross-sectional web-based survey was distributed to all Community Rehabilitation and Support Workers (CRSWs) working at Neami National (n = 471), an Australian community mental health service. The validated questionnaire assessed participants’ self-rated oral health knowledge and confidence (7 questions); their perceived barriers (9 questions) and attitudes (5 questions) to oral health promotion; and their oral health promotion practices (7 questions). Differences in responses between groups were analysed using Chi-square, Fisher’s exact and Mann–Whitney U tests. Logistic Regression Analysis served to explore the probability of providing oral health support to mental health consumers.

**Results:**

A total of 141 CRSWs were included in this study, achieving a response rate of 30 percent. Roughly two-fifths (39.0%) of participants had oral health training in the previous 12-months. The majority of CRSWs (89.3%) believed (‘Agreed’ or ‘Strongly agreed’) that mental health support workers have a role to play in promoting oral health. However, less than half (44.0%) of CRSWs practiced oral health promotion activities when working with mental health consumers. When asked about barriers to promoting oral health, ‘lack of consumer interest’ was the most prevalent issue. CRSWs who had oral health training were over three-times (OR 3.5, 95% CI 1.25–9.83, p = 0.017) more likely to provide oral health support. Results showed the provision of oral health support was most strongly associated with self-rated knowledge and confidence (OR 4.089, 95% CI 1.629–10.263, *p* = 0.003) and attitudes to oral health promotion (OR 3.906, 95% CI 1.77–8.65, *p* = 0.001).

**Conclusion:**

The results of this study suggest that mental health support workers who have more positive attitudes to oral health promotion and who have higher self-rated oral health knowledge and confidence are more likely to provide oral health support in their professional role. Training for community mental health professionals is essential to build confidence and skills to promote oral health for mental health consumers.

## Background

The global burden of mental illness is rising [1, 2]. It is estimated that one-in-five adults around the world experienced a common mental disorder (i.e. anxiety, mood and substance use disorders) in the previous twelve months [2]. The severity of mental illness is based on the diagnosis, the intensity and duration of symptoms, and the degree of associated disability [3]. Severe mental illness includes lower-prevalence disorders such as schizophrenia and other psychotic disorders; however, it also includes more severe and disabling mood and anxiety disorders. Approximately 2–3% of Australian adults are currently living with a severe mental disorder [3].

People living with severe mental illness (SMI) experience persistently poor physical health outcomes and excess mortality [4]. In addition, oral health problems are prevalent among people living with SMI, with high rates of tooth loss and untreated dental decay [5–11]. Fear, stigma, cost of care, and communication issues are barriers to people living with SMI accessing timely and appropriate oral health care [11–14]. Psychotropic medications (e.g. antidepressants, anti-anxiety drugs and antipsychotics) can cause dry mouth and increase the risk of oral diseases [15]. The behavioural risk factors for oral diseases (e.g. poor diet, smoking and unsafe alcohol consumption) are also associated with other chronic health conditions (e.g. diabetes, cardiovascular disease, respiratory diseases, cancer) [16]. The majority of oral diseases and conditions are preventable; [17] therefore, more should be done to develop targeted oral health promotion programs to improve oral health outcomes for people living with SMI.

Specialised community mental health services provide a range of rehabilitation and support care to people living with SMI in the community [18]. Community mental health professionals are ideally placed to initiate conversations with mental health consumers about oral health, to offer basic oral health information and to facilitate consumer access to oral health care [19, 20]. To date, little has been done to improve oral health promotion practices in community mental health settings.

The Smile for Health program is a partnership between the Melbourne Dental School at the University of Melbourne and Neami National, an Australian community mental health service [21]. The program aims to increase capacity to provide oral health support for people living with SMI. Neami identified oral health as an unmet need for Neami consumers and since 2013 oral health has been one of the Neami health promotion priority areas. Qualitative research involving focus groups and semi-structured interviews with Neami consumers identified the ‘value of receiving oral health support’ from community mental health professionals [20]. In this present study, we sought to explore the self-rated oral health knowledge and confidence, attitudes and professional practices of Neami Community Rehabilitation and Support Workers (CRSWs).

## Methods

### Study design and setting

This anonymous cross-sectional web-based survey was approved by the Human Research Ethics Committee at the University of Melbourne (ethics ID 1544795) and by the Neami Research and Evaluation Committee. As of November 2015, there were 471 CRSWs working at Neami across five Australian states. According to the Neami Research and Evaluation Committee survey response rates at Neami are generally between 35 and 50%; therefore, in order to maximise the number of respondents, all CRSWs were invited to participate. Based on the number of CRSWs and the anticipated response rate of 35–50%, the expected sample size was 165–236 respondents.

Community Rehabilitation and Support Workers were invited to participate in this study via an email, which included a copy of the plain language statement (PLS) explaining the study and a link to the questionnaire on Survey Gizmo, a web-based survey platform [22]. To maintain their confidentiality, the email invitation was distributed by the Neami Research and Evaluation Officer (who was part of the project advisory group). The web-based survey also contained a copy of the PLS and advised CRSWs that by choosing to continue with the survey they were providing consent for the use of their data. CRSWs who did not currently provide direct consumer support were excluded from the study. Survey steps outlined by Dillman were followed (e.g. notifying potential participants prior to the invitation email being sent and sending a reminder email one week after the invitation) to maximise the response rate [23]. Data was collected between November 2015 and March 2016.

### Survey tool

The questionnaire used was purpose-designed for this study. The development of the survey questions began with a review of the dental and mental health literature [24–31]. The knowledge, confidence and attitudes questions were derived from previous Australian studies [32, 33]. A panel of experts (which consisted of three academics from the University of Melbourne with expertise in oral health and health promotion and six Neami representatives with expertise in mental health (including consumers, health promotion and research staff and senior management) worked collaboratively to determine the validity of the questions included in the survey tool. The questionnaire included sections assessing: oral health knowledge (27 Likert items and 11 Yes/No/Don’t know questions); self-rated oral health knowledge and confidence (7 Likert items); perceived barriers to oral health promotion (9 Likert items); attitudes to oral health promotion (5 Likert items) and oral health promotion practices (7 questions). In the section on oral health promotion, participants responded ‘Yes’ or ‘No’ when asked if they practised oral health-related activities with Neami consumers; this question was followed by six sliding-scale questions asking about the proportion of consumers provided with different types of oral health support (i.e. discussing oral health, providing oral health care advice, providing oral health education materials, referring consumers to dental services, making dental appointments for consumers, attending dental appointments with consumers).

In compliance with Neami Research and Evaluation Committee requirements, limited demographic data was collected from participants. The questionnaire was piloted with a small group of Neami staff who had previously worked as a CRSW. The questionnaire was then revised and approved by the project advisory group. Reliability and validity of the revised scales was reviewed. Cronbach’s alpha was used to determine the internal reliability. The reliabilities of the three scales used in the present study (i.e. self-rated knowledge and confidence, barriers to oral health promotion and attitudes to oral health promotion) were found to be in the range of 0.72 to 0.84. Validity of the scale was assessed through criterion and face validity.

In mid-October 2015, second year Bachelor of Oral Health (BOH) students from the University of Melbourne delivered short, 30-min, face-to-face oral health training sessions to staff at twelve Neami sites in Victoria, Australia. The planning and delivery of oral health education sessions is one of the intended learning outcomes of the health promotion subject in which the BOH students were enrolled; students designed and prepared for their presentations as a class exercise, with support from the lead author (RMcG) who was the subject coordinator. Although these training sessions were not planned as part of this study, it was decided to consider their impact as part of the data analysis. Therefore, results were compared between:

1. CRSWs working in Victoria and CRSWs working outside Victoria,
2. CRSWs who had worked at Neami for two years or more and CRSWs who had worked at Neami for less than two years, and
3. CRSWs who had completed oral health training in the previous 12-months and CRSWs who had not.

### Data analysis

Univariate statistics were used to describe the respondents’ characteristics (i.e. located in Victoria or the rest of Australia, years worked at Neami, and participation in oral health training in the previous twelve months) and responses to each of the survey questions. Bivariate analysis of nominal and ordinal variables was performed using Chi-Square and Fisher’s Exact tests. In order to perform further analyses, mean scores were calculated from Likert items (responses were scored from 1 through to 5, dependent upon the direction of the responses) and results for grouped Likert items were combined into sum variables including: ‘self-rated knowledge and confidence’, ‘attitudes to oral health promotion’ and ‘perceived barriers to oral health promotion’; Mann–Whitney U tests were conducted when analysing these variables.

Logistic Regression Analysis was performed to determine the effects of selected independent variables, namely being located in Victoria, working at Neami for longer than two years, and having participated in oral health training (coded as: Yes/No) and the sum scores of ‘self-rated oral health knowledge and confidence’, ‘perceived barriers to oral health promotion’, and ‘attitudes to oral health promotion’ variables on CRSWs oral health promotion practice (i.e. whether they do or do not promote oral health when working with mental health consumers). As this was an exploratory study, all *p *values < 0.05 were considered significant. Data manipulation and analysis was performed using SPSS version 23.0.

## Results

A total of 166 CRSWs responded to the web-based survey. However, 24 respondents partially completed the survey (for unknown reasons) and another respondent was disqualified as they did not currently provide direct consumer support. This meant that the total number of surveys included in the analysis was 141, giving a final response rate of 30 percent. Roughly half of respondents were from Victoria (n = 69, 48.9%) and about three-quarters (n = 104, 73.7%) had commenced working with Neami within the previous 24-months (Table 1). Two-fifths (n = 55, 39.0%) had oral health training in the preceding year, most of whom were Victoria-based (n = 49, 89.1%).

**Table 1:** Profile of a sample of Australian community rehabilitation and support workers (n = 141), by state

		NSW	QLD	SA	VIC	WA	Total
		N (%)	N (%)	N (%)	N (%)	N (%)	N (%)
Location		23	9	26	69	14	141
		(16.3)	(6.4)	(18.4)	(48.9)	(9.9)	(100.0)
Years worked at Neami	< 1	12	5	8	25	7	57
		(55.2)	(55.6)	(30.8)	(36.2)	(50.0)	(40.4)
	1–2	4	1	8	30	4	47
		(17.4)	(11.1)	(30.8)	(43.5)	(28.6)	(33.3)
	2–3	4	2	4	7	1	18
		(17.4)	(22.2)	(15.4)	(10.1)	(28.6)	(12.8)
	3–4	2	1	5	3	1	12
		(8.7)	(11.1)	(19.2)	(4.3)	(7.1)	(8.5)
	> 4	1	–	1	4	1	7
		(4.3)	–	(3.8)	(5.8)	(7.1)	(5.0)
Oral health training***	Yes	1	2	3	49	–	55
		(4.3)	(22.2)	(11.5)	(71.0)	–	(39.0)
	No	22	7	23	20	14	86
		(95.7)	(77.8)	(88.5)	(29.0)	(100.0)	(61.0)
Aware of oral health programme**	Yes	13	5	11	57	5	91
		(56.5)	(55.6)	(42.3)	(82.6)	(35.7)	(64.5)
	No	10	4	15	12	9	50
		(43.5)	(44.4)	(57.3)	(17.4)	(64.3)	(35.5)

NSW = New South Wales, QLD = Queensland, SA = South Australia, VIC = Victoria, WA = Western Australia

**Fisher’s Exact test *p* value < 0.01

***Fisher’s Exact test *p* value < 0.001

### Attitudes to oral health promotion

The results from the ‘attitudes to oral health promotion’ section (Table 2) indicate that the vast majority of respondents ‘Agreed^’^ or ‘Strongly agreed’ that: supporting consumers with their oral health needs should be part of Neami’s work (n = 122, 86.6%); and that people living with SMI should be encouraged to visit a dental practitioner regularly (n = 136, 97.2%). Close to half (n = 64, 45.4%) did not feel (responded ‘Disagree’ or ‘Strongly disagree’) that the inclusion of oral health promotion into Neami’s routine work would leave less time to address clients’ mental health-related issues. Nearly two-thirds (n = 80, 57.1%) also disagreed that oral health education should only be provided by an oral health professional. In fact, most CRSWs believed that mental health professionals have a role to play in promoting oral health (n = 126, 89.3%).

**Table 2:** A sample of Australian Community Rehabilitation and Support Workers’ (n = 141) attitudes to oral health promotion

	Strongly disagree	Disagree	Neutral	Agree	Strongly agree
	N (%)	N (%)	N (%)	N (%)	N (%)
Supporting consumers with their oral health needs should be part of Neami’s work	–	2	17	73	49
	–	( 1.4)	(12.1)	(51.8)	(34.8)
People living with SMI should be encouraged to visit an oral health professional regularly	–	–	4	75	61
	^−^	–	(2.8)	(53.2)	(43.3)
Mental health support workers have a role to play in promoting oral health	–	1	14	80	46
	^−^	(0.7)	(9.9)	(56.7)	(32.6)
Including oral health promotion will leave less time to address consumers’ MH needs	18	46	48	19	10
	(12.8)	(32.6)	(34)	(13.5)	(7.1)
Oral health education should only be provided by an oral health professional	16	64	42	11	7
	(11.3)	(45.4)	(29.8)	(7.8)	(5)

Almost all (n = 69, 95.8%) of respondents outside Victoria believed they have a role to play in promoting oral health, which was significantly higher than in Victoria (n = 57, 82.6%) (FET, *p* = 0.016). There were no significant differences in sum attitudes to oral health promotion scores dependent upon years worked at Neami or participation in oral health training.

### Barriers to oral health promotion, as perceived by CRSWs

Many respondents (n = 93, 65.9%) believed that barriers exist to oral health promotion and that lack of consumer interest is the most important issue (n = 82, 58.2%) (Table 3). Almost two-fifths of CRSWs felt that staff have insufficient knowledge about oral health (n = 53, 37.5%) and that lack of time (n = 53, 37.8%) is a barrier. While some CRSWs did not feel staff had insufficient knowledge about dental services (n = 47, 33.6%) or insufficient access to oral health resources (n = 59, 41.8%), for others these barriers were of concern (39.0% and 26.2% respectively). Most CRSWs (n = 125, 88.7%) did not think that lack of support from Neami management, or attitudes of oral health professionals were barriers to promoting oral health (n = 117, 83.0%).

**Table 3:** A sample of Australian community rehabilitation and support workers’ (n = 141) perceived barriers to oral health promotion within Neami

	Strongly disagree	Disagree	Neutral	Agree	Strongly agree
	N (%)	N (%)	N (%)	N (%)	N (%)
Oral health low on Neami agenda	8	47	61	23	2
	( 5.7)	(33.3)	(43.3)	(16.3)	(1.4)
Not enough time when working with consumers	3	42	42	44	9
	(2.1)	(29.8)	(29.8)	(31.2)	(6.4)
Consumers’ lack of interest in oral health	3	24	32	65	17
	(2.1)	(17)	(22.7)	(46.1)	(12.1)
Staff have insufficient knowledge about oral health	3	36	49	48	5
	(2.1)	(25.5)	(34.8)	(34)	(3.5)
Staff have insufficient knowledge about dental services	5	42	37	49	7
	(3.5)	(29.8)	(26.2)	(34.8)	(5)
Staff have insufficient access to oral health resources	14	45	45	32	5
	(9.9)	(31.9)	(31.9)	(22.7)	(3.5)
Insufficient support from management	18	57	50	14	2
	(12.8)	(40.4)	(35.5)	(9.9)	(1.4)
Attitudes of oral health professionals	5	45	67	19	5
	(3.5)	(31.9)	(47.5)	(13.5)	(3.5)
There are no barriers	37	56	40	5	3
	(26.2)	(39.7)	(28.4)	(3.5)	(2.1)

CRSWs in Victoria were less likely to believe that they had insufficient knowledge about oral health compared to those outside Victoria (FET, *p* = 0.002). In addition, fewer in Victoria thought that lack of access to oral health resources was a barrier to oral health promotion (FET, *p* = 0.002). In considering training, less of those who had oral health-related professional development thought that lack of: knowledge about oral health (FET, *p* < 0.001), knowledge about dental services (FET, *p* = 0.035), access to oral health resources (FET, *p* < 0.001), or support from management (FET, *p* = 0.037) were barriers to oral health promotion. Sum ‘perceived barriers to oral health promotion’ scores (Table 4) were significantly higher for those outside Victoria compared to Victoria-based CRSWs (U = 1,854.5, z = -2.602, *p* = 0.009). Results also indicate that those who had participated in oral health training believed there were less barriers to providing oral health promotion than those who had no training (U = 3,220, z = 3,622, *p* < 0.0001).

**Table 4:** Comparing a sample of Australian Community Rehabilitation and Support Workers’ (n = 141) sum scores for perceived barriers to oral health promotion

	N	Mean	SE	*p* Value
VIC	69	2.93	0.065	0.009
Non-VIC	72	3.17	0.056	
> 2 years	37	2.99	0.094	0.469
< 2 years	104	3.07	0.049	
OH training	55	2.85	0.068	< 0.0001
No OH training	86	3.17	0.053	
All CRSWs	141	3.05	0.044	

VIC = Victoria

Mann–Whitney U tests

### Oral health promotion practices

Participants were asked if they actively promote oral health when working with consumers (Table 5). Over half of respondents (n = 79, 56.0%) indicated that they did not currently engage in any oral health promotion activities. Analysis showed that a significantly greater proportion of CRSWs in Victoria (52.2%) practised oral health promotion compared to those in other Australian states (36.1%) (χ^2^(1) = 3.960, *p* = 0.040). In addition, CRSWs who had participated in oral health training were significantly more likely to provide oral health support (60.0%), compared to those who had no training (33.7%) (χ^2^(1) = 9.403, *p* = 0.002). While more of those who had worked at Neami for longer than two years practised oral health promotion (48.6% compared to 42.3% for those who had worked there for a shorter time-period), this result was not statistically significant (p > 0.05).

**Table 5:** Proportion of a sample of Australian community rehabilitation and support workers (n = 141) who practise oral health promotion

	Yes	%	No	%	Total	*p* Value
VIC	36	52.2	33	47.8	69	0.040
Non-VIC	26	36.1	46	63.9	72	
> 2 years	18	48.6	19	51.4	37	0.317
< 2 years	44	42.3	60	57.7	104	
OH training	33	60.0	22	40.0	55	0.002
No OH training	29	33.7	57	66.3	86	
All CRSWs	62	44.0	79	56.0	141	

VIC = Victoria

Mann–Whitney U tests

Self-rated knowledge and confidence was significantly higher for those who had participated in oral health training, compared to those who had no training (U = 1,497.5, z = − 3.680, *p* < 0.0001). CRSWs with higher self-rated knowledge and confidence (e.g. in discussing oral health with consumers, supporting consumers with their oral health needs, referring consumers to oral health services) were more likely to practice oral health promotion (U = 1.515, z = − 3.894, *p* < 0.0001). Those who provided oral health support also had more positive attitudes to oral health promotion (U = 1,705, z = − 3.110, *p* = 0.002) and perceived fewer barriers to including oral health promotion in their work (U = 2,956, z = 2.113, *p* = 0.035).

CRSWs who practised oral health promotion (n = 62, 44.0%) responded to questions about the types of support they provided. They were most likely to: discuss oral health with consumers (97%), refer consumers to dental services (97%) and support consumers when attending dental appointments (94%). However, the vast majority also provided support to improve daily oral health care (89%) and contacted dental clinics to make appointments for consumers (84.0%).

The binomial logistic regression results are presented in Table 6. The model was statistically significant (χ ^2^(5)  = 34.633, *p* < 0.0001); it explained 29.2% of the variance on CRSWs oral health promotion practice, and correctly classified 67.4% of cases. CRSWs who had oral health training were three and a half times more likely to provide oral health support than those who had no training (OR 3.50, 95% CI 1.246–9.829). Additionally, the provision of oral health promotion and support was associated with self-rated knowledge (OR 4.089, 95% CI 1.629–10.263) and attitudes to oral health promotion (OR 3.906, 95% CI 1.765–8.648). Location and years worked at Neami were not found to have a multivariate effect.

**Table 6:** Logistic regression analysis of factors associated with practising oral health promotion in a sample of Australian community rehabilitation and support workers (n = 141)

	B	SE	*p* Value	OR	Or 95% CI	
Location	− 0.013	0.503	0.979	0.987	0.368	2.645
Years at Neami	0.412	0.439	0.348	1.510	0.638	3.574
Oral health training	1.253	0.527	0.017	3.500	1.246	9.829
Self-rated knowledge and confidence	1.408	0.470	0.003	4.089	1.629	10.263
Attitudes to oral health promotion	1.363	0.405	0.001	3.906	1.765	8.648
Perceived barriers to oral health promotion	0.710	0.465	0.127	2.034	0.817	5.060
Constant	− 12.276	3.343	0.0001			

χ^2^ (5, n = 141) = 34.663, *p* < 0.0001, Nagelkerke R^2^ 29.2%, 67.4% of cases

## Discussion

In this study, we explored the oral health promotion practices of Australian community mental health professionals. We found that participation in oral health training, higher self-rated knowledge and confidence, and more positive attitudes to oral health promotion were associated with increased provision of oral health support when working with people living with SMI. This study provides valuable initial findings on oral health promotion practices in a community mental health setting.

The survey response rate (30%) was lower than expected and lower than that achieved in other surveys of mental health professionals [26, 34, 35]. The majority of participants were from Victoria or New South Wales, which reflected the geographic distribution of Neami services and staff. Although Neami was experiencing a period of rapid growth in 2015, we did not anticipate that three-quarters of respondents would have worked with the organisation for less than two-years. Mental health services are known to have high rates of staff turnover [36]; this suggests a need for regular oral health training opportunities for community mental health professionals to ensure they are skilled and confident in providing oral health support to mental health consumers.

We were encouraged to find that, in general, Neami CRSWs generally had positive attitudes to oral health promotion. They recognised that they have a role to play in supporting consumers’ oral health and believed oral health promotion should be delivered in community mental health settings. Other studies with health professionals from a range of disciplines also found participants to be supportive of oral health promotion programs targeting the population groups they work with, and indicated the need for appropriate strategies to integrate oral health promotion activities into routine service delivery [28, 29, 37–45]. There is strong evidence that non-dental professionals (e.g. aged care professionals, midwives, nurses) can play an important role in promoting oral health [38, 46–48]. There is a need to translate the positive attitudes of community mental health professionals into improvements in the provision of oral health support for people living with SMI.

Despite recognition of the importance of oral health promotion for people living with SMI, the results of our study highlight a number of barriers to effective implementation (e.g. consumers’ lack of interest in oral health, staff having insufficient knowledge about oral health and dental services, and lack of time when working with consumers). A qualitative study of Western Australian community mental health workers’ views on oral health identified similar barriers to this present study [41]. Our results indicate that CRSWs who rated their oral health knowledge and confidence higher perceived fewer barriers to oral health promotion. This confidence in their oral health knowledge may mean they are better able to problem-solve and overcome potential barriers to oral health promotion as they arise. Problem-solving is an important skill in health promotion and it creates sustainability in capacity building approaches [49]. Future oral health training for community mental health professionals should focus on enhancing their capacity to support consumers to address their personal barriers to achieving optimal oral health.

The number of survey respondents who indicated that they practice oral health promotion was lower than Neami management expected (44%), particularly as oral health had been a Neami health promotion priority area since 2013. The CRSWs who answered that they do practise oral health promotion were more likely to discuss oral health with their clients than they were to contact a dental clinic or refer clients to dental services. While it seems reasonable to advise community mental health professionals to refer consumers to dental clinics, it is important to understand that this may not align with ‘recovery oriented’ practice. Neami service delivery is underpinned by the Collaborative Recovery Model (CRM), which is built upon a foundation of consumer autonomy and promotes the individual’s responsibility in managing his or her own health needs and lifestyle choices [50]. This means CRSWs would be more likely to support consumers to make dental appointments for themselves, rather than to do it for them.

The promotion of oral health by non-dental professionals is supported by evidence as an effective approach. [25, 38, 42, 51–55] However, it is essential to understand how community mental health services are delivered in order to design contextually appropriate oral health promotion strategies to align with models of care (e.g. the CRM) to integrate into existing organisational structures.

As with all studies, there may be some limitations to the generalisability of the findings. Although the sample size was lower than anticipated, it was representative of the target population and large enough to conduct the required statistical analysis. While we would have preferred to conduct this survey prior to CRSWs in Victoria completing oral health training, we addressed this in the analysis by comparing results between CRSWs who had oral health training in the previous 12-months and CRSWS who had not had training. This led to us finding that oral health training was significantly associated with the provision of oral health support, which actually strengthened the study findings.

The results from the knowledge section of this survey, which found oral health training was significantly associated with oral health knowledge, have been previously published in a professional association members-only journal [56]. This present paper expands on the association between participants’ self-rated oral health knowledge and confidence and their oral health promotion practices when working with mental health consumers. Additionally, it presents information about Australian community mental health professionals’ attitudes to oral health promotion, their perceived barriers to promoting oral health and their oral health promotion practices.

## Conclusion

This cross-sectional survey provides important new information about oral health promotion practices in a community mental health setting. Our results suggest that community mental health professionals believe they have a role to play in promoting oral health and that training increases their capacity to provide oral health-related support to people living with SMI. The findings from this exploratory study were used to design strategies, as part of the Smile for Health Program, to build capacity within Neami to promote oral health for people living with severe mental illness.

## Data Availability

Ethics approval was granted on the basis that only researchers involved in the study could access the data. Raw data have been securely stored at the Melbourne Dental School, The University of Melbourne. The minimum retention period is five years. Supporting documents are available on request from the corresponding author.

## Abbreviations

CRSWs: Community Rehabilitation and Support Workers
SMI: severe mental illness
